# Innovative Ultrasound Criteria for the Diagnosis of Adenomyosis and Correlation with Symptoms: A Retrospective Re-Evaluation

**DOI:** 10.3390/biomedicines12020463

**Published:** 2024-02-19

**Authors:** Anna Biasioli, Matilde Degano, Stefano Restaino, Margherita Bagolin, Francesca Moro, Francesca Ciccarone, Antonia Carla Testa, Pantaleo Greco, Giovanni Scambia, Giuseppe Vizzielli, Lorenza Driul, The Udine Hospital Endometriosis Group

**Affiliations:** 1 Clinic of Obstetrics and Gynecology, Santa Maria della Misericordia University-Hospital, Azienda Sanitaria Universitaria Friuli Centrale, Piazzale Santa Maria Della Misericordia, 15, 33100 Udine, Italy; restaino.stefano@gmail.com (S.R.); giuseppe.vizzielli@uniud.it (G.V.); lorenza.driul@uniud.it (L.D.); 2 Department of Medicine, Università degli Studi di Udine, Via Palladio, 8, 33100 Udine, Italy; marghe899@gmail.com; 3 Dipartimento Scienze della Salute della Donna, del Bambino e di Sanità Pubblica, Fondazione Policlinico Universitario Agostino Gemelli, IRCCS, 00168 Rome, Italy; francesca.moro@policlinicogemelli.it (F.M.); francesca.ciccarone@policlinicogemelli.it (F.C.); antoniacarla.testa@policlinicogemelli.it (A.C.T.);; 4 Istituto di Clinica Ostetrica e Ginecologica, Università Cattolica del Sacro Cuore, 00168 Rome, Italy; 5 Department of Medical Sciences, Institute of Obstetrics and Gynecology, University of Ferrara, 40121 Ferrara, Italy; grcptl@unife.it

**Keywords:** adenomyosis, ultrasound, diagnosis, pelvic pain, infertility, abnormal uterine bleeding

## Abstract

The 2022 Delphi revision of the MUSA (Morphological Uterus Sonographic Assessment) criteria for the ultrasound diagnosis of adenomyosis divides the ultrasound signs for diagnosis into direct and indirect ones, considering the presence of at least one direct sign as a mandatory criterion. This study aimed to reclassify the patients referred to the Pelvic Pain specialist outpatient clinic of the Gynecological Clinic of Udine according to the new criteria, evaluating the number of overdiagnoses and the possible correlation between the direct and indirect signs and the patients’ symptoms. 62 patients affected by adenomyosis were retrospectively recruited. The patients were then re-evaluated by ultrasound and clinically. At least one direct sign of adenomyosis was found in 52 patients, while 16% of the population examined did not present any. There was no statistically significant difference between patients presenting direct signs and those presenting none for the symptoms considered. According to the new criteria, 16% of the patients examined were not affected by adenomyosis; applying the new consensus to symptomatic patients could increase false negatives. In a population of symptomatic patients, the diagnosis of adenomyosis is still highly probable even without direct ultrasound signs, given the clinical symptoms and having ruled out other causes of such symptoms.

## 1. Introduction

Adenomyosis is a benign uterine pathology characterized by the presence of endometrial glands and stroma within the myometrium, leading to hypertrophy and hyperplasia of the smooth muscle cells of the myometrium itself. It was first described by the German pathologist Karl von Rokitansky in 1860 [1]. Adenomyosis can present as a diffuse or focal lesion (adenomyoma) and may involve the inner or outer myometrium. The topographic distribution of adenomyotic lesions is variable: in most cases, the disease appears to originate from the endometrial-myometrial interface, with a subsequent centrifugal extension towards the external myometrium. The depth of myometrial infiltration is also variable, from cases limited to the innermost myometrium to those involving the entire myometrial thickness. It is a frequent pathology with an estimated prevalence of 20–30% in the female population [2]. However, the true prevalence is not exactly well known. This has been attributed to both the facts that the disease often presents asymptomatically and the lack of standardized diagnostic criteria. It is often associated with endometriosis, but also the prevalence of this association varies widely in the literature [3,4,5,6]. For some authors, the prevalence of adenomyosis in endometriosis patients is 80–90%. Other studies have found adenomyosis in less than half of patients presenting with endometriosis, or even an absence of a relationship between adenomyosis and endometriosis [2,7]. Adenomyosis has a major impact on the quality of life of affected women due to the very disabling symptoms. These are mainly pelvic pain, heavy menstrual bleeding, association with infertility, and obstetrical complications (even in naturally conceived pregnancies) such as preeclampsia, preterm delivery, fetal growth restriction, and postpartum hemorrhage [5,8,9,10]. 

The heavy menstrual bleeding is the main symptom of this pathology and can be attributed to various causes: an increase in uterine volume, an increased vascularity, improper uterine contractions, and/or an increase in estrogen and prostaglandin production [11]. Regarding pelvic pain (that can be considered in terms of dysmenorrhea, dyschezia, dysuria, and periovulatory pain), it was demonstrated that it worsens with the increasing depth and extent of adenomyosis invasion into the myometrium, as well as with the number of adenomyotic foci. Finally, adenomyosis can also have a negative impact on fertility: the presence of a dysregulation of myometrial structure can lead to an alteration in myometrial peristalsis, and to an altered endometrial function. It has been hypothesized that factors such as defects in decidualization (which reduce endometrial receptivity), activation of local and systemic inflammatory pathways, increased production of prostaglandins, and alterations in placentation may also be involved [8,12,13]. 

Exacoustos et al. [14] proposed a study that aimed to correlate the type and degree of adenomyosis with symptoms and fertility, through an ultrasound study of affected patients. What emerged from this study was a difference between focal and diffuse adenomyosis concerning age, menstrual bleeding, infertility, and miscarriages. However, a correlation between the severity of symptoms and the extent of the disease has not been demonstrated. Specifically, the study highlighted how diffuse adenomyosis was more frequent in elderly women with heavy menstrual bleeding than in those with focal adenomyosis. Furthermore, they demonstrated a higher rate of infertility and miscarriage in focal adenomyosis. Severe diffuse adenomyosis also appears to be correlated with severe dysmenorrhea and menorrhagia.

The difficulty in the epidemiological framing of adenomyosis is also linked to the fact that, until the recent past, the diagnosis was mainly performed by surgery [12]. The definitive diagnosis of adenomyosis is based on the histological study of the uterus which allows us to highlight the ectopic endometrial tissue in the myometrium: hysterectomy is still the gold standard for the diagnosis [15]. However, nowadays the characteristics of the pathology have been identified and described in magnetic resonance imaging (MRI) and ultrasound. This created the opportunity to investigate pathogenesis, molecular expressions, clinical impact, and outcomes of medical and procedural interventions [11,16]. Magnetic resonance imaging is an accurate and non-invasive technique usually used as a second-level exam as it is more expensive and less available than ultrasound. However, it is a more reproducible test, with sensitivity, specificity, and positive and negative prediction values of 77.5%, 92.5%, 83.8%, and 89.2%, respectively. Its use has grown significantly in recent decades [2,6,17]. 

Currently, the first-line method for the diagnosis of adenomyosis is transvaginal ultrasound (TVUS), which has been shown to be considerably more accurate than the transabdominal technique [2,18,19,20]. It represents a direct, minimally invasive, cost-effective, widely available, and non-contraindicated examination. Another advancement has been the introduction of three-dimensional ultrasound (3D-TVUS), which is particularly useful in studying adenomyosis, especially for evaluating the junctional zone [18,19,20,21]. Exacoustos et al. compared the characteristics of adenomyosis detectable with two- (2D) and three-dimensional (3D) examinations and related them to the histopathological characteristics of the junctional zone (JZ) and of the myometrium [22]. The presence of myometrial cysts was the most specific feature of 2D-TVUS, while heterogeneous myometrium was the most sensitive feature. The markers of 3D-TVUS with high sensitivity and best accuracy were the infiltration and distortion of the JZ [22]. 

In 2015, the MUSA (Morphological Uterus Sonographic Assessment) consensus was published to standardize terminology and develop accurate and uniform criteria for the ultrasound diagnosis of adenomyosis [21]. In 2018, the same group recommended a uniform classification and reporting system to be used for providing a detailed description of the morphology and extent of adenomyosis, including terms, definitions, and measurements. According to this consensus, just a positive or negative diagnosis of adenomyosis was not enough. Instead, a detailed description of all the lesions identified by ultrasound had to be made. In this way, the following characteristics could be described: the localization of the disease in the affected uterine wall (anterior, posterior, left lateral, right lateral, or fundic); whether the lesion is focal or diffuse; the presence or absence of myometrial cysts; the degree of involvement of the depth of the myometrium (limited to the internal portion, invasion of the entire body of the uterus, or invasion reaching the serosa); the volume of the uterus (<25%, 25–50%, >50%); and the size of the lesions [17]. 

After the 2018 publication of the MUSA criteria, a pilot study highlighted that the inter-rater agreement when using the MUSA features to describe ultrasound images of adenomyosis was poor both among highly experienced and moderately experienced raters, probably due to the unclear definitions of the ultrasound characteristics [23]. Therefore, in 2021, the MUSA criteria were re-evaluated in a new consensus, based on the review made by a panel of expert sonographers, to refine the diagnosis of this pathology using a Delphi procedure [24]. In this new consensus, the ultrasound characteristics suggestive of adenomyosis proposed in MUSA 2015 are divided into direct (cysts, hyperechoic islands, echogenic sub-endometrial lines, and buds) and indirect ones (asymmetrical thickening, fan-shaped shadowing, translesional vascularity, globular uterus, irregular junctional zone, and interrupted junctional zone). The diagnosis of adenomyosis is made when at least one direct sign is present. Direct signs indicate the presence of ectopic endometrial tissue in the myometrium while indirect signs are secondary to the presence of endometrial tissue in the myometrium, such as muscular hypertrophy (globular uterus), or artifacts (e.g., shadowing). Furthermore, experts have agreed that evaluating the junctional zone (JZ) is highly useful, especially in cases of diagnostic uncertainty. These signs have very high specificity (86–98% for myometrial cysts, 83–95.5% for hyperechoic islands, and 78% for hyperechoic lines) but low sensitivity (47–55%, 12–54%, and 51%, respectively). Indirect signs, on the other hand, are easier to detect, especially in the case of advanced disease, as they are associated with myometrial hypertrophy. However, if they are present in the absence of direct signs, a diagnosis of adenomyosis cannot be made; these cases are defined as uncertain. In cases of uncertainty, the junctional zone can be examined using 3D-TVUS. If the junctional zone appears to be intact, the diagnosis of adenomyosis can be ruled out [24].

This study aimed to retrospectively reclassify the patients referred to the specialist outpatient clinic for Pelvic Pain at the Gynecological Clinic of Udine, according to the criteria of the new consensus, assessing any diagnostic discordance and overdiagnoses. This reclassification has potentially important implications, with obvious repercussions in terms of clinical management, and use of resources, and with an economic, health, and psychological impact. A secondary objective, on the other hand, was to study the correlation existing between direct and indirect ultrasound signs, according to the revised classification, and the clinical symptoms of the patients by evaluating AUB (abnormal uterine bleeding), pelvic pain, and infertility.

## 2. Materials and Methods

Patients diagnosed with adenomyosis, referred to the specialist outpatient clinic for Pelvic Pain of the University of Udine Gynecological Clinic from April 2017 to April 2022, were retrospectively evaluated. 

The inclusion criteria were reproductive age (from menarche to menopause) and diagnosis of adenomyosis. 

The following exclusion criteria were also applied: ongoing estrogen-progestin/progestin therapy at the time of the enrollment, associated endometriosis, previous surgery for endometriosis, multiple uterine myomas, absence of ultrasound video archiving, or 3D volume of the uterus. 

The initial diagnosis, made between 2017 and 2022, was made considering clinical symptoms and transvaginal ultrasound (TVUS) examination according to the MUSA 2015 criteria [21]. These involved the recognition of a series of ultrasound characteristics which, however, were not divided into direct and indirect and were not configured in a precise diagnosis scheme. The ultrasound features considered to be typical of adenomyosis were as follows: the presence of myometrial cysts, hyperechogenic islands, echogenic sub-endometrial lines and buds, asymmetrical thickening, fan-shaped shadowing, translesional vascularity, irregular junctional zone, and interrupted junctional zone. All included patients had at least one of these signs, not considering the distinction between direct/indirect ones. The diagnosis was made with a Samsung WS80A (Suwon-si, Republic of Korea) ultrasound machine by the same operator, who is an expert in the ultrasound diagnosis of adenomyosis and endometriosis (A.B. MD). Ultrasound examination was conducted with both two-dimensional and three-dimensional techniques for the systematic evaluation of the pelvis, uterus, and ovaries, the diagnosis of adenomyosis, and the exclusion of other pathologies. 

As regards the exclusion of patients with endometriosis, we excluded from recruitment all the patients with a concomitant diagnosis of endometriosis carried out histologically or clinically/ultrasound (the evaluation was always performed by the same expert operator).

The following information was collected for each patient at the time of diagnosis: age, parity, height, weight, BMI, associated pathologies, and year of diagnosis. Furthermore, pain symptoms were documented on an NRS (Numerical Rating Scale) [25] from 1 to 10, in terms of dysmenorrhea, chronic pelvic pain, dyspareunia, dyschezia, dysuria, and finally the characteristics of the menstrual cycle in terms of regularity of rhythm, duration, and quantity, with the presence or absence of abnormal uterine bleeding (AUB) or heavy menstrual bleeding (HMB) [12,14,26,27]. 

All ultrasound videos and images collected between 2017 and 2022 were then re-evaluated by the same operator, and the cases were reclassified according to the criteria of the new MUSA consensus [24]. The patient was considered to have the disease if at least one direct sign was present. If there were indirect signs only, the diagnosis was made considering the characteristics of the junctional zone, which, if altered, allows the diagnosis to be confirmed [24]. A correlation was therefore performed between the presence of direct and indirect signs, and the clinical presentation, such as painful symptoms (dysmenorrhea, lumbar pain, pain during ovulation, dyspareunia, dyschezia, and dysuria), presence or absence of menstrual cycle abnormalities in terms of AUB, and infertility.

This study was approved by the Institutional Review Board of Udine with protocol n°138/23.

Statistical analysis was conducted using Chi-square tests to verify differences and associations between the studied variables. Variables were expressed in terms of mean ± standard deviation (SD), while categorical variables were expressed in terms of frequency and percentage. Statistical significance was considered for a *p*-value < 0.05.

## 3. Results

We retrospectively included in the study 62 patients with adenomyosis who were referred to the specialist outpatient clinic for Pelvic Pain between April 2017 and April 2022. The mean age of the patients (±SD) was 43 (±8.65) years, the mean height was 164 (±6.09) cm, the mean weight was 65.86 (±11.92) kg, the mean BMI was 24.49 (±4.25), and the mean uterine volume was 222.51 (±148.98) cm^3^ (Table 1).

The most represented sign corresponds to the hyperechoic islands, present in 42/62 (66%) cases, followed by myometrial cysts in 25/62 (40%), and echogenic sub-endometrial lines in 10/62 (16%). Concerning indirect signs, the most represented one consists of the presence of fan-shaped shadowing, in 43/62 (69%) patients, followed by globular uterus in 33/62 (53%), asymmetrical thickening in 30/62 (48%), junctional zone anomalies in 22/62 (35%), and translesional vascularity in 9/62 (14%).

Of the 62 analyzed, 52 (84%) presented at least one direct sign. These patients, according to the new consensus [13], were therefore those who were classified as suffering from adenomyosis. Among the patients with direct signs, 30/52 (58%) had only one ultrasound direct sign, 19 patients (36%) had two direct signs, and 3 (6%) had all three direct criteria (Table 2).

Among the 30 out of 52 patients (58%) presenting only one ultrasound sign, 21 presented only hyperechoic islands, 5 had only myometrial cysts, and 4 presented only hyperechoic lines. Among the 19 patients with two direct signs (36%), 16 had cysts and hyperechoic islands, 1 had cysts and lines, and 2 had hyperechoic islands and lines.

**Figure 1:**
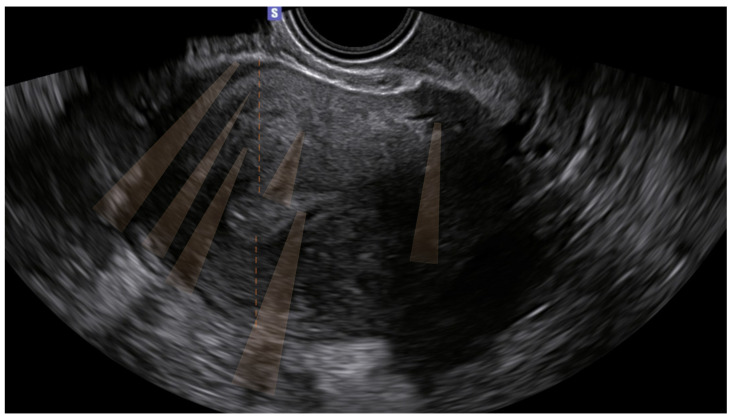
Ultrasound image of the uterus of one of the ten patients who presented only indirect signs (shadowing, highlighted in orange in the figure). The patient was symptomatic (dysmenorrhea).

The 10 remaining patients (16%) presented only indirect signs, and the diagnosis was uncertain. Of these, none had junctional zone anomalies (Table 3) (Figure 1). 

Concerning the clinical symptoms, within the 62 patients, 52/62 (84%) had pelvic pain, 10/62 (16%) had infertility, and 5/62 (8%) had AUB. In the population with only direct signs, i.e., out of the total of 52 patients, 43/52 (83%) had pain, 4/52 (8%) had AUB, and 8/52 (15%) reported infertility. Specifically, the most common symptom is dysmenorrhea, which is encountered in 45 out of 62 patients (72%), with no statistically significant difference between the two groups. Following this, the most frequent symptoms are dyspareunia and periovulatory pain, present in 45% and 40% of the total patients, respectively.

Among the patients with uncertain diagnosis (10 patients), 9/10 (90%) had pain, 2/10 (20%) had infertility, 1/10 (10%) had AUB, and there were no cases of asymptomatic patients (Table 4).

Clinically, there was no statistically significant difference in symptomatology between the patients with direct signs and patients without direct signs, except for periovulatory pain.

## 4. Discussion

Adenomyosis diagnosis is traditionally confirmed by histopathologic examination of the uterine specimen [17,28,29]. MUSA (Morphological Uterus Sonographic Assessment) ultrasound criteria [24] allowed clinicians to standardize the diagnosis of adenomyosis, allowing for a clinical diagnosis and greater uniformity, useful for clinics and research, and opening a new epidemiological scenario [18]. The investigators rightfully stated that the criteria cannot be used alone to decide on treatment, but rather, they need to be validated in future studies evaluating the relationship between the sonographic features and the clinical outcomes. 

The revised criteria state that in the absence of direct features, ultrasound examination is not conclusive for the presence of adenomyosis, and that may be useful to avoid overdiagnosis. However, in the paper, there is no distinction between symptomatic and asymptomatic patients. An effective approach to the diagnosis of adenomyosis cannot ignore the use of non-invasive diagnostic tools, the profile of risk factors, and the clinical and imaging aspects. A recent review on this topic [30] concluded that there is no consensus between classification systems and their ability to correlate with clinical findings, and the authors hope for increased research in imaging and clinical correlations, having found only 16 articles that matched these inclusion criteria.

Moreover, in another recent review [31], directed to correlate ultrasound features with clinical manifestations of adenomyosis and to discuss diagnostic methods for predicting disease severity, the authors reported that the lesion thickness, diffuse or internal adenomyosis, and focal adenomyosis may be associated with increased risks of symptoms, and that two ultrasound markers (i.e., the presence of heterogeneous myometrium and myometrial cysts) appear to be the most associated criteria. They concluded that there is currently no consensus that symptom severity can be predicted based on ultrasound features.

In this study, we demonstrated that patients referred to the specialist outpatient clinic for Pelvic Pain at Udine Gynecological Clinic and diagnosed as suffering adenomyosis according to MUSA 2015 consensus, were reclassified as patients with uncertain diagnosis in 16% of cases, according to the criteria established by the new consensus [24]. 

A strong point of this paper is the homogeneity of the population affected by adenomyosis only, having excluded patients with concomitant endometriosis and fibromatosis. However, some forms of endometriosis cannot be diagnosed solely by clinical/ultrasound examination, but with the criteria used by an expert sonographer, endometriosis can be excluded with reasonable certainty. Furthermore, all patients who had a histological diagnosis of endometriosis were excluded. Another strength of this work is that the ultrasound evaluations were consistently made by the same experienced operator, both in the initial assessment and in the subsequent re-evaluation according to the new criteria. The equipment used was also consistently the same, allowing for a uniform assessment that was not dependent on the operator or the machine. Certainly, between 2017 and 2022, the operator’s expertise may have increased, but this does not seem to affect the study results, as the starting expertise level was already that of an experienced sonographer. Furthermore, the subsequent re-evaluation of images (both for the diagnosis of adenomyosis and the exclusion of other pathologies) was carried out in a short timeframe, enabling a consistent standard for image re-evaluation.

The limitation of the study, however, is that it is a retrospective, single-center study with a limited number of patients. The number of patients in both groups is indeed too small to draw significant conclusions about the analysis of symptoms. Furthermore, a parameter not considered in this study is the uterine extension of adenomyosis, a parameter that could be significant concerning the clinical symptoms. All these limitations, however, could serve as a basis for further future studies with a larger number of patients. As a final note, although major confounding factors related to symptoms (like endometriosis and fibromatosis) have been excluded, there are other causes, such as hypercontractility, hyperesthesia, or endocrine factors, which cannot be entirely ruled out for conditions like dysmenorrhea.

Most patients with direct signs of adenomyosis (57.7%) had only one direct sign. The most frequent sign was, in accordance with the literature [24], hyperechoic islands, followed by myometrial cysts, and echogenic sub-endometrial lines. Direct ultrasound signs indicate the presence of endometrial glands and stroma beyond the sub-endometrial layer; indirect features, on the other hand, express a myometrial reaction consisting of hypertrophy. They can be present even in the absence of direct signs but make the diagnosis of adenomyosis uncertain. Direct signs have high specificity and lower sensitivity: the authors report specificity values of 86–98% for myometrial cysts, 83–95.5% for hyperechoic islands, and 78% for echogenic sub-endometrial lines; sensitivity is 47–55%, 12–54%, and 51% for the three characteristics, respectively [22,24,32,33,34,35]. 

In our case series, 10 patients do not show any direct signs, irregularities, or interruption of the junctional zone. Therefore, 16% of the examined population, according to the new indications in the literature, would not be affected by adenomyosis or have an uncertain diagnosis. Since we are dealing with symptomatic patients, however, we cannot assume that we have eliminated a proportion of ultrasound overdiagnosis. Indeed, the initial hypothesis was that the new criteria would be more restrictive than the previous ones, leading to a lower number of false positives. However, in this population of symptomatic patients, the diagnosis is still highly probable given the clinical symptoms and having already ruled out other causes of such symptoms. Therefore, applying the new consensus to symptomatic patients could increase false negatives, making the method less sensitive.

## 5. Conclusions

This study aims to find a correlation between direct and indirect ultrasound signs and the patient’s clinical presentation, which is the secondary aim of our analysis. Comparing the two groups, no clinically significant differences (except for periovulatory pain) were found between patients with at least one direct sign and those without direct signs. 

In the case of highly symptomatic patients, the presence of only indirect signs could be linked to the difficulty of finding the direct ones, since these are obscured by the extent of an abnormal myometrial reaction. Indeed, indirect signs are specifically those linked to the myometrial reaction. In other previous works, the connection between diffuse adenomyosis (versus focal), more evident symptomatology (especially AUB), and the age of patients has been highlighted [14]. On the other hand, our hypothesis needs to be verified in further studies, which are currently not present in the literature. 

With the limitations described, we can conclude that the application of the criteria proposed by the new consensus in the examined population, composed of symptomatic patients, is not more restrictive than the application of the old criteria. In fact, the diagnosis of adenomyosis must consider not only imaging but also clinical aspects. While in asymptomatic patients, the presence of only indirect (and not direct) criteria excludes the diagnosis of adenomyosis and helps reduce overdiagnosis, this does not seem to be equally true in symptomatic patients, as the ultrasound data should not be the sole diagnostic criterion.

## Figures and Tables

**Table 1:** Characteristics of the population.

Demographic Data	Mean Value	SD
Age	43.19	±8.65
Height	164 cm	±6.09
Weight	65.9 kg	±11.92
BMI	24.5 kg/m^2^	±4.25
Mean uterine volume at TVUS examination	222.5 cm^3^	±148.98

**Table 2:** Distribution of the ultrasound direct signs.

Patient Group	N° of Patients (%)	Ultrasound Sign (n° of Patients)
Patients with 1 direct sign		Hyperechoic islands (21)
	30 (58%)	Myometrial cysts (5)
		Echogenic sub-endometrial lines (4)
Patients with 2 direct signs		Cysts and islands (16)
	19 (36%)	Islands and lines (2)
		Cysts and lines (1)
Patients with 3 direct signs	3 (6%)	

**Table 3:** Distribution of the ultrasound indirect signs.

Indirect Sign	N (%)
Fan-shaped shadowing	43/52 (69%)
Globular uterus	33/52 (53%)
Asymmetrical thickening	30/52 (48%)
Translesional vascularity	9/52 (14%)
JZ anomalies	22/52 (35%)

**Table 4:** Correlation between ultrasound signs and symptoms.

	Total Patients (62)	Patients with Direct Signs (52)	Patients without Direct Signs (10)	
	N (%)	N (%)	N (%)	*p*-Value
AUB	5 (8%)	4 (8%)	1 (10%)	>0.05
Infertility	10 (16%)	8 (15%)	2 (20%)	>0.05
Dysmenorrhea	45 (72%)	37 (71%)	8 (80%)	>0.05
Lumbar pain	9 (14%)	9 (17%)	0 (0%)	>0.05
Pain during ovulation	25 (40%)	18 (35%)	7 (70%)	0.0367
Dyschezia	12 (19%)	9 (17%)	3 (30%)	>0.05
Dysuria	5 (8%)	3 (6%)	2 (20%)	>0.05
Dyspareunia	28 (45%)	22 (42%)	6 (60%)	>0.05

## Data Availability

The data presented in this study are available on request from the corresponding author. The data are not publicly available due to privacy restrictions.
